# Current state of follow-up care for patients with Hymenoptera venom anaphylaxis in southwest Germany: Major impact of early information

**DOI:** 10.1007/s40629-017-0046-7

**Published:** 2018-01-16

**Authors:** Manisha Manmohan, Sabine Müller, Michèle Myriam Rauber, Frank Koberne, H. Reisch, Joachim Koster, Richard Böhm, Martin Messelken, Matthias Fischer, Thilo Jakob

**Affiliations:** 1 Department of Dermatology, Allergy Research Group, Medical Center, University of Freiburg, Freiburg, Germany; 2 Department of Dermatology and Allergology, University Medical Center Giessen, Justus Liebig University, Gaffkystraße 14, 35392 Giessen, Germany; 3 Dermatology and Plastic Surgery Institute, Cleveland Clinic, 9500 Euclid Ave., 44195 Cleveland, OH USA; 4 Emergency Medical Response Center Freiburg, St. Joseph’s Hospital, Freiburg, Germany; 5 Emergency Medical Response Center Bad Krozingen, University Heart Center Freiburg—Bad Krozingen, Bad Krozingen, Germany; 6 Department of Anesthesia, Intensive Care and Emergency Medicine, Alb Fils Kliniken GmbH, Klinik am Eichert, Göppingen, Germany

**Keywords:** Insect venom allergy, Anaphylaxis, Guideline, Follow-up care

## Abstract

**Background:**

Up to 3.5% of the population experience anaphylactic reactions in response to Hymenoptera stings. Current guidelines are in place for the diagnostic workup and follow-up care of patients with Hymenoptera venom anaphylaxis (HVA). However, little is known about the degree of implementation of the recommendations and patient attitudes toward the recommendations in the general patient population.

**Methods:**

For the analysis of the follow-up care in real life, a retrospective questionnaire-based study was conducted in unselected patients who had received treatment from an emergency medical response team for HVA, as documented in records of three regional Medical Emergency Response Centers.

**Results:**

From over 125,000 cases, a filtered list of 1895 patients that coded for anaphylaxis was generated and examination of paper records identified 548 patients with a documented insect sting anaphylaxis. Patients were sent a standardized questionnaire addressing different aspects of diagnostics and follow-up care. Almost 40% of the patients did not receive a referral to an allergist at the emergency center, over 50% did not consult an allergy specialist at any time after the index sting, 25% did not receive any form of diagnostic workup, over 30% did not receive any information about venom immunotherapy (VIT) as treatment option, and only 50% were eventually started on VIT. Emergency medication was prescribed in 90% of the cases, 77% including an adrenalin auto injector, of which 47% were expired at the time of the survey. Patients who were informed about diagnostic and treatment options early during the index event, i. e., during the stay in the emergency department, displayed a higher rate of referral to an allergist (70% vs. 17%), higher rate of diagnostic workup (88% vs. 59%), and a higher rate of initiation of VIT (89% vs. 64%), as compared to patients who did not.

**Conclusion:**

Our results demonstrate that there are missed opportunities for secondary and tertiary prevention of anaphylaxis due to insect venom allergy and that early information on required diagnostics and treatment options has a major impact on the degree of proper follow-up care in line with current guideline recommendations.

## Introduction

Insect venom is the most common cause of anaphylaxis in adults in Germany as documented by the German Anaphylaxis Register [1]. Systemic anaphylactic reactions to bee or wasp stings (hymenoptera venom allergy, HVA) are present in up to between 0.4–3.5% of the general population [2–9]. These acute reactions often present as medical emergencies and the patients should receive the emergency medical treatment appropriate for an anaphylactic reaction. After the patient has recovered from the index event, patient treatment should continue. Long-term prophylactic treatment includes patient education on exposition prophylaxis, emergency medications for self-treatment, and allergen specific immunotherapy (venom immunotherapy, VIT).

Guidelines have been developed for the diagnosis and treatment of patients with bee or wasp venom allergies by the German, Austrian, and Swiss Societies for Allergy and Clinical Immunology in cooperation with the German Societies for Dermatology, Pediatric Allergy and Environmental Medicine, and Pediatric Medicine as well as the Association of German Allergists [10]. Among others, these guidelines provide specific recommendations for management in the emergency situation, diagnostic workup, prescription and use of emergency self-medications and for treatment with VIT. In addition an updated guideline for the management of insect venom allergy has been published by the insect venom allergy interest group of the European Academy of Allergy and Clinical Immunology [11].

While detailed recommendations for patient management exist, to our knowledge, there are no studies that evaluate the reality of patient care after the index event in Germany. Prior studies in the United States have found a wide range of rates for referral and follow-up with allergists. A single center study of patients with anaphylaxis found a rate of 31–38% [12, 13], studies of patients with HVA have ranged between 14 and 20% [14, 15]. These studies either used medical record review exclusively or insurance claims databases. Little is known about rates of VIT among patients with HVA under real-life conditions.

The objective of our study was to evaluate the follow-up care that patients receive with regard to emergency medications, allergist visits, diagnostics and VIT. A patient population with a history of insect sting anaphylaxis—that was otherwise unselected—was identified from the medical records of three regional emergency medical centers and queried directly via a written questionnaire in order to avoid selecting for patients who had received follow-up care.

## Methods

### Study design and setting

A retrospective review of emergency medical records was conducted for patients evaluated by the emergency medical teams of three centers, Freiburg (FR), Bad Krozingen (BK), and Göppingen (GP). Patients included were sent a questionnaire via regular mail. The University of Freiburg and Baden-Württemberg State Chamber of Physicians Ethic Commissions approved the study protocol.

### Patient selection

The set of medical records made available for examination was different at each emergency center. In FR the years 2001–2013 were examined, 2001–2014 in BK, and 2004–2011 in GP.

From over 125,000 cases, a filtered list of 1895 emergency medical records that coded for anaphylaxis were identified and examined for the German words for “sting”, “insect”, “insect venom”, “bee,” “wasp,” “hornet,” and “bumble bee.” Adults (aged >18 years) and children (aged <18 years) were included if they had required the emergency medical response team due to an insect sting. A total of 548 patients were identified with documentation of insect sting.

Final inclusion in the study was dependent on the patient returning the questionnaire and self-reporting that they had been stung by a hymenoptera (not another insect or animal) and had a reaction with systemic symptoms.

### Questionnaire

All patients identified as having a potential anaphylactic reaction due to hymenoptera venom were sent a questionnaire by mail with a stamped return envelope. In addition to general demographic information, the questionnaire explored four main areas: type and severity of anaphylactic reaction (7 questions), treatment in the emergency situation (5 questions), disease management, diagnostics, and emergency medications (17 questions), and finally, VIT (10 questions).

### Statistics

Patient questionnaires were then analyzed using IBM SPSS statistical software. Descriptive statistics and custom tables were primarily used. When appropriate, Chi-squared tests were used for determining dependencies.

## Results

### Demographic and clinical characteristics

Of the 548 patients sent questionnaires, a total of 148 questionnaires were returned, for an overall response rate of 27%. After the questionnaires were analyzed to determine that the respondent had indeed had HVA, we were able to include a total of 126 patients. Our final patient cohort was comprised of 75 patients from FR, 29 from BK, and 22 from GP. A summary of patient demographics is presented in Table 1.

**Table 1:** Demographics of patient population

Demographic	*n* = 126
*Gender*	*No. (Percent)*
Female	64 (50.8)
Male	62 (49.2)
*Insurance*	
Public	94 (74.6)
Private	28 (22.2)
Other	2 (1.6)
No reply	2 (1.6)
*Age (years)*	*Median (IQR)*
	54 (45–63)

*IQR* interquartile range

### Characteristics of index event

Based on the symptoms reported, patients were classified into four severity grades according to the criteria proposed by Ring and Messmer [16]. There were 7 patients with a grade 1 (mildest) reaction, 30 patients with grade 2, 81 patients with grade 3, and 8 patients with grade 4 reactions. Over 70% of respondents were not aware of their allergy at the time of their emergency intervention, and 80% were transported to a hospital for further monitoring after stabilization of the initial reaction.

### Post index outcomes

#### Initial follow-up

During the index event, 55% of the patients received recommendations for follow-up with information on required diagnostics and/or treatment options, 37% did not receive any recommendation, while 8% did not remember or did not answer whether they received information during the index event. Almost 70% of patients did not receive an allergy identification card during the initial treatment and over 40% of patients reported not receiving a prescription for emergency medications during their acute treatment. Only 17% received both written and verbal information on preventing future insect stings, while 35% did not receive any information on sting avoidance.

#### Prescription of emergency medication for self treatment

Current guidelines mandate the prescription of oral glucocorticoids, oral antihistamines, and epinephrine auto-injectors (EAI).

Emergency medications were well prescribed, with 90% of patients reporting that they received a prescription for emergency medications at some point during the follow-up care, while only 60% received this prescription during the initial treatment. Over half of patients who received medications were instructed in how to use them, but only 23% actually received hands-on practice. Only 77% of patients with emergency medication received a prescription for an EAI and at the time of survey 47% of EAIs were expired. Sixty one percent of patients reported that their physician did not check their emergency medications during follow-up visits.

**Fig. 1:**
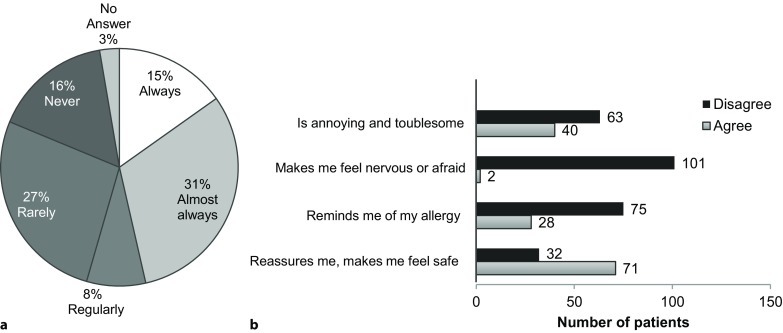
**a** Frequency of patients who carry emergency medications. **b** Patient attitudes toward emergency medications

After filling their prescription 32% of patients reported to have used their emergency medications at least once. A substantial percentage (43%) of patients either rarely or never carried their medications with them (Fig. 1a). The top reason for not carrying emergency medications was forgetfulness, with 50 patients naming it as a reason. Thirty two patients said that auto-injector size was a reason that they did not carry the EAI. Patient attitudes toward carrying emergency medications are summarized in Fig. 1b.

#### Referral to allergist and diagnostics performed

After the initial emergency treatment, almost 40% of patients did not receive a referral to see an allergist, and 15% did not see any physician for follow-up (8 patients did not answer). Only 46% reported seeing an allergist for follow-up.

**Fig. 2:**
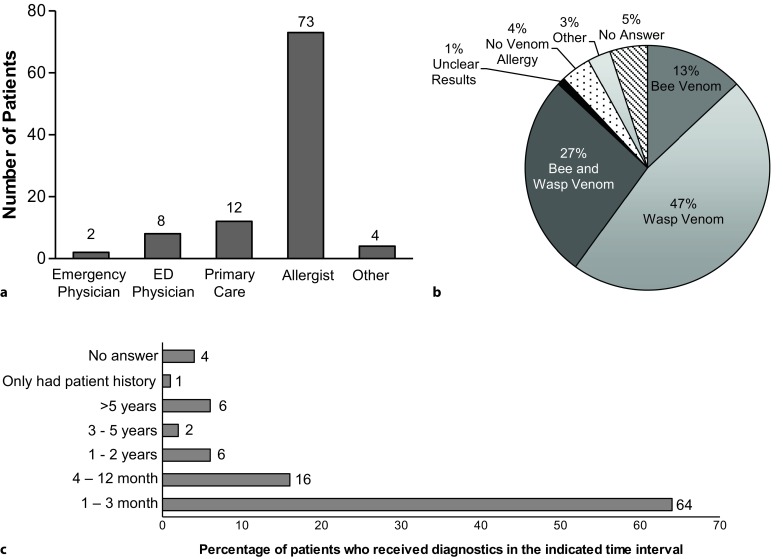
**a** Physician(s) with whom diagnostic testing was undertaken. **b** Results of diagnostic testing. **c** Time interval from index event to diagnostics performed

Almost one quarter (28/126) of patients did not receive any diagnostic testing any time after the index event. As presented in Fig. 2a, of the patients who received diagnostics (*n* = 98), 73 patients reported receiving diagnostics from an allergist. The next most common providers were primary care physicians, with 12 patients reporting.

Fig. 2b demonstrates the allergy diagnoses received. Eighty one patients reported to have received test results that confirmed an allergy to hymenoptera venom. This is 64% of the entire patient population, and 86% of those patients who received diagnostic testing. As seen in Fig. 2c, of those receiving diagnostic testing, 80% were diagnosed within one year of their index event.

#### Venom immunotherapy

Seventy percent of patients received information about VIT, and 50% started the treatment. Of the 62 patients who did not start VIT, almost half received neither diagnostics nor information about VIT as a treatment option. Six patients received diagnostics and were diagnosed with HVA, but reported not receiving any information on VIT. Fourteen patients were diagnosed with HVA and received information about VIT but did not start VIT for various reasons. Patients were asked to write-in their reasons for not undergoing VIT. A summary is presented in Table 2.

**Table 2:** Commencement of venom immunotherapy (VIT) and the status with regard to diagnostic testing, diagnoses, information regarding VIT of patients who did not begin VIT, and reasons given for not starting VIT

*Patient population (n =* *126)*	*No. (%)*
Currently receiving VIT	16 (13)
Completed VIT	38 (30)
Started but did not finished VIT	8 (6)
Will start VIT	1 (1)
No answer	1 (1)
No VIT	62 (49)
*Patients not starting * *VIT * *(n =* *62)*	*No. (%)*
Did not receive diagnostics, received information	5 (8)
Did not receive diagnostics, did not receive information	27 (44)
Diagnosed with HVA, received information	14 (23)
Diagnosed with HVA, did not receive information	6 (10)
Other	8 (13)
*Reason given by patients for not starting VIT*	*No.*
Unnecessary	3
Not interested	2
Only had one reaction	5
Not an option	1
Too much effort	4
Takes too long	3
Did not want to be hospitalized	1
Expensive	1
Risk to high	2
Not effective	2
Have heard about negative experiences	2

*HVA* Hymenoptera venom anaphylaxis

#### Factors influencing medical follow-up care

Twenty-seven percent of all patients reported receiving follow-up care at University Hospital Medical Center Freiburg (UKF), and another 13% reported receiving follow-up at another secondary or tertiary center.

As detailed in Table 3, all patients who received follow-up at a tertiary center received diagnostic testing compared with 92% of those receiving treatment at a secondary center. All patients who saw an allergist in private practice also received diagnostics, compared with 51% of those patients who saw a non-allergist physician. Patients treated at a tertiary center reported having received information on VIT in 97% of the cases, as compared to 92% of the patients treated at secondary centers. Eighty-three percent of patients who saw an allergist in private practice received information regarding VIT, compared with only 34% of those who saw a non-allergist physician.

**Table 3:** Patients receiving diagnostic workup and information regarding venom immunotherapy (VIT) by follow-up provider classification

Follow-up provider	No.	Received diagnostic workup (%)	Received VIT information (%)
Tertiary (allergist)	38	100	97
Secondary (allergist)	12	92	92
Primary (allergist)	18	100	83
Non-allergist provider	35	51	34

**Fig. 3:**
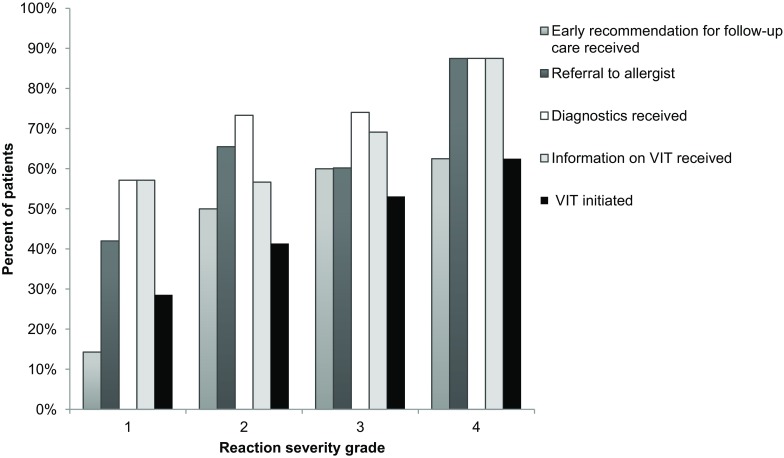
Influence of index sting severity grade according to Ring and Messmer [16] on patient follow-up care with regard to early recommendation for follow-up received, referral to allergist, diagnostics performed, information regarding venom immunotherapy (VIT) received, and VIT initiated

Although the sample size is small, particularly in the grade 1 and grade 4 groups, we observed a trend that increased reaction severity was associated with a higher rate of referral to an allergist, a higher percentage of patients receiving diagnostics and information on VIT and a higher rate of patients starting VIT. Despite this trend, among the severe reactors (grade 3 and 4; *n* = 89) only 61% were referred to an allergist, 75% received proper diagnostics, 70% received information on VIT, and only 54% were started on VIT (Fig. 3).

Finally, there were factors that did not affect follow-up treatment. There was no statistically significant difference in patient cohorts (FR, BK, GP) and follow-up treatment, nor did private vs. public insurance influence follow-up. Gender also did not play a role in patients receiving follow-up treatment (data not shown).

To get an overall picture of the treatment path of each patient, a tree diagram was constructed with the most pertinent end points: 1. received a recommendation for follow-up (during the acute treatment), 2. saw an allergist, 3. received emergency medications for home use, 4. received diagnostic testing, 5. result of diagnostic testing (positive for bee and/or wasp venom or not), 6. informed about VIT, 7. received VIT. For simplicity, if a patient did not respond to a question or did not remember they were grouped in as giving a negative response, with the exception of the topic “allergist visit” where they were given their own category.

Finally, it was striking that receiving an early recommendation for follow-up was a strong predictor of patients actually seeing an allergist, receiving diagnostic testing, receiving information regarding VIT and starting VIT. Of the 69 patients who received an early recommendation, 96% (66/69 patients) received a prescription for emergency medication, 70% (48/69) saw an allergist, 88% (61/69) received diagnostic testing and 89% of those that were tested positive (47/53) went on to receive VIT (Fig. 4a and b).

**Fig. 4:**
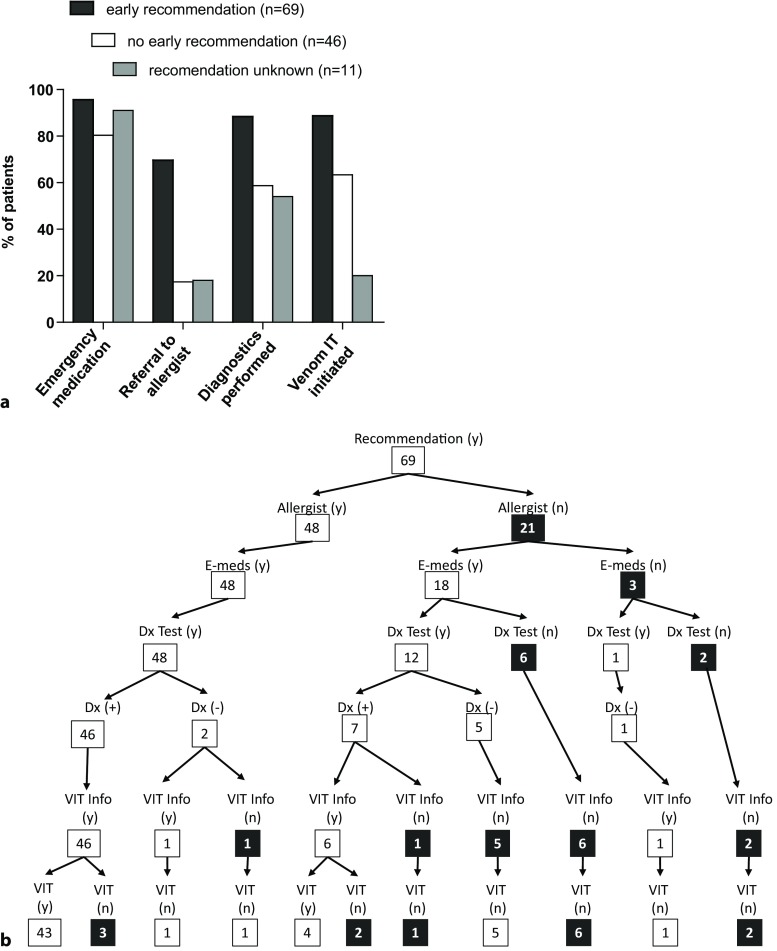
**a** Rate of emergency medication prescribed, rate of referrals to an allergist, rate of diagnostics performed and rate of venom immunotherapy (VIT) initiated in patients who received an early recommendation for follow-up during the acute treatment phase (*n* = 69) as compared to patients who did not receive this recommendation during acute treatment (*n* = 46), or did not remember/did not answer if a recommendation was received during acute treatment (*n* = 11). **b–d** Tree diagram of treatment paths reported by patients who received an early recommendation for follow-up (*n* = 69) (**b**), by patients who did not receive an early recommendation for follow-up (*n* = 46) (**c**) or did not remember/did not answer if a recommendation was received during acute treatment (*n* = 11) (**d**). Relevant endpoints: (1) early recommendation for follow-up received (**b**) not received (**c**), unknown (**d**)

**Fig. 4 (Continued):**
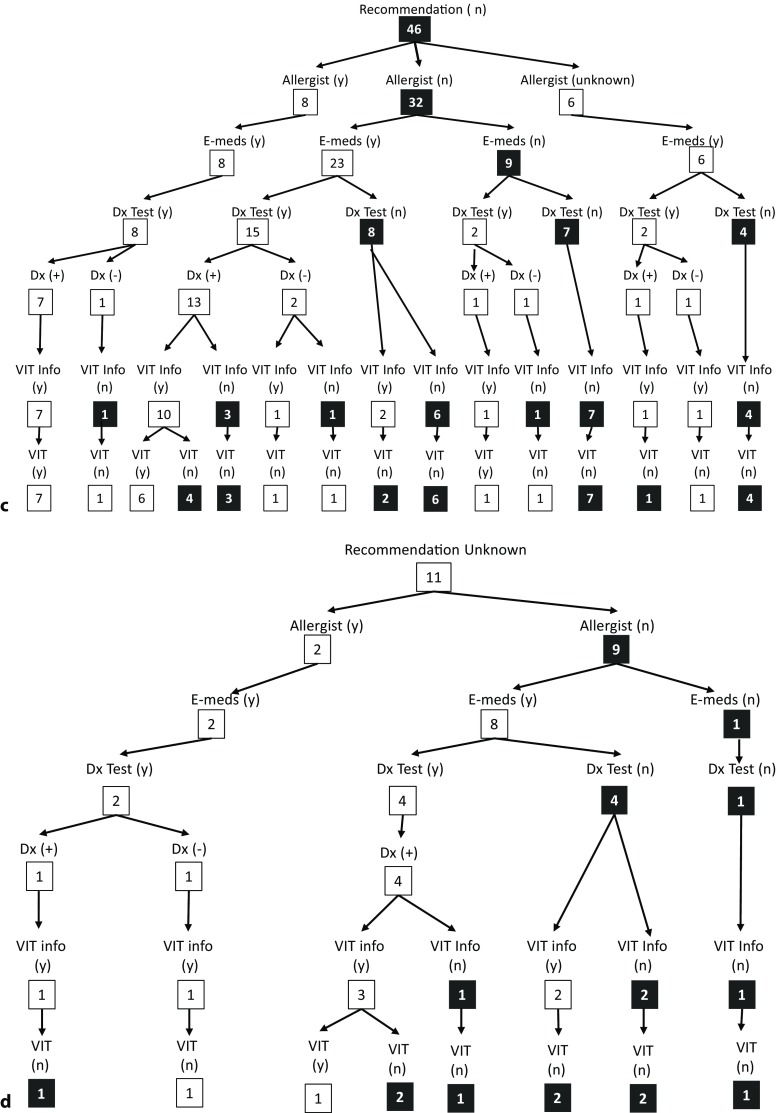
(2) referral to an allergist received, (3) prescription for emergency medications received, (4) diagnostic testing performed, (5) results of diagnostic testing (positive for bee and/or wasp venom or not), (6) information about VIT as treatment option received, (7) VIT initiated. The treatment/follow-up that is not in line with the current guideline recommendations is highlighted in black. (*Recommendation* follow-up recommendation during acute treatment; *Allergist* referral to allergist received; *E-meds* received a prescription for emergency medications; *Dx Test* received diagnostic testing; *VIT Info* informed about venom immunotherapy as treatment option; *VIT* venom immunotherapy initiated; (y) = yes; (*n*) = no; (+) = positive; (–) = negative)

In contrast, of those who did not receive an early recommendation for follow-up (*n* = 46), 80% (37/46) received emergency medication, 17% (8/46) saw an allergist, 59% (27/46) received diagnostic testing, and 64% of those that were tested positive (14/22) went on to receive VIT (Fig. 4a and c). Eleven patients did not remember or did not answer the question whether they received early recommendation for follow-up care during the acute treatment phase (Fig. 4d). In this group 90% (10/11) received emergency medication, 18% (2/11) saw an allergist, 54% (6/11) received diagnostic testing, and 20% of those that were tested positive (1/5) went on to receive VIT (Fig. 4a and d).

## Discussion

In designing our study, the primary goal was to gain a comprehensive understanding of the follow-up care patients received during and after an anaphylactic insect sting reaction, including their attitudes toward the preventive care options and how the received follow-up care may have differed from the current guidelines. This study is unique in that patients were directly queried about their sting event and the follow-up care they received. This enabled us to capture allergist visits and treatment that was initiated after the acute treatment, as well as to ask patients about their attitudes regarding therapy options, and to gain insight into the reasoning behind the decision to not undergo treatment, and to understand the extent that patients continue to receive preventive care in the years following the index event. It was particularly important to capture follow-up treatment initiated by the primary care physician because the primary care physician often provides referrals in Germany. Prior studies that exclusively used medical records and chart reviews were limited to the chart documentation, and use of insurance claims databases enabled prior studies to accurately capture EAI prescriptions and allergist visits, but did not allow for inquiry into patient attitudes. These studies also did not investigate long-term care with regard to VIT [14, 15].

Emergency medications were initially well prescribed, with 77% of patients receiving EAIs. However, at the time of survey half of the EAIs had expired. This finding is comparable to that of Fisher et al. who reported that 54% of emergency kits contained expired medications and that 60% of EAIs were expired [17]. Furthermore, 61% of patients also reported that their physician did not check their emergency medications during follow-up consultations. Closer follow-up of prescriptions by primary care physicians could help to increase the number of patients with current emergency medications.

Previous studies have shown that the rate of referral to an allergist upon discharge from the emergency department is low [12, 14, 18]. These studies did not capture referrals from primary care physicians. The overall rate of allergist visits that we found (46%), while still not ideal, is much higher than those reported by earlier studies, in which only 14–23% of patients followed up with an allergist. It is important to note that these studies looked at a defined length of time and were conducted using medical and insurance records [15, 19]. The increased rate of allergist visits found in our study may be due to our method of asking patients to self-report and the self-selection of patients responding to our questionnaire. The allergist visit is key to receiving proper diagnostics and by implication when tested positive the recommendation for VIT. In addition, Campbell et al. [13] reported that in patients with anaphylaxis to an unknown trigger, the allergy visit resulted in the identification of the trigger in 32% of the cases.

Overall, 62% of patients reported receiving information on insect sting avoidance. This is much higher than the Clark et al. multicenter average of 20%; however, they also found a wide variance of between 0–69% among centers [14]. Informing the patient about how to avoid future stings is a low cost preventative measure that could be easily implemented in the form of a hand out.

In contrast to an earlier study, which found that patients with more severe episodes (cardiorespiratory failure) were less likely to receive preventative care after the index event with an odds ratio of 0.50 for any preventative care [15], we found the opposite to be true, with patients with grade 3 and 4 reactions more likely to receive VIT than patients with grade 1 and 2 reactions. However, in our study only 60% of patients with a grade 4 reaction received VIT. When indicated, VIT has a success rate of up to 95% [20–22]. Somewhat surprising was that 14 patients, 17% of those with a diagnosis of insect venom allergy, did not start VIT, despite receiving information about the therapy. An additional 6 patients reported receiving a diagnosis, but no information about VIT. For patients in Germany, there is a relatively low direct financial cost of receiving VIT; however, the treatment is time intensive, requiring multiple visits. This is reflected in the reasons that patients gave for not undergoing VIT, with 8 patients giving reasons related to the effort and commitment involved, compared with only one patient saying that VIT was too expensive. Another 5 patients reported that they did not undergo VIT because they only had one reaction.

Several studies have shown that on a sting challenge, 30% of children and as many as 60% of adults with a history of insect venom anaphylaxis will have a systemic reaction if they do not receive VIT [22–25]. Studies have also shown that 30% of patients with systemic reactions to stings had experienced at least 2 systemic sting reactions [26, 27]. Additional patient education or a new approach to educating patients on insect venom allergy and the benefits of VIT could be beneficial in increasing the number of patients choosing VIT. Given that VIT can only be initiated if patients receive proper diagnostic testing [28], it is important for patients to follow-up not only with their primary care physician, but also with an allergist. Only half of patients who followed up with only a primary care physician received diagnostics, while following up with an allergist in a primary, secondary, or tertiary center essentially guaranteed that the patient received diagnostics, thus, enabling them to receive VIT if indicated.

One of the more striking findings of our study was the predictive value of an early recommendation for further follow-up during the acute treatment phase. Seventy percent of patients who received an early recommendation reported a visit with an allergist, compared with only 18% of those who did not. A key strategy to increase rates of VIT could be to increase awareness of VIT efficacy in emergency departments and in primary care providers.

It should be noted that an analysis of VIT for insect venom allergy in the UK suggested that VIT is only cost effective if patients are stung frequently (e. g., beekeepers), or if quality of life improvement is considered. While there is some debate surrounding the cost effectiveness of VIT, there is agreement that VIT becomes cost effective when quality of life is improved as a result [29, 30]. There are also several studies that have shown that a less severe systemic reaction is a risk factor for future severe reactions [31, 32]. Current German and European guidelines recommend VIT for all patients reactions of grade 2 severity or higher and for patients with a grade 1 reaction if they have any other risk factors or if their quality of life has been negatively impacted [10, 11, 33].

If the guidelines were followed, all patients would have been eligible to receive a diagnostic workup, and those tested positive would have been eligible to receive VIT. In contrast to guideline recommendations, only 60 patients with grade 2 or higher received VIT, which demonstrates a clear deficit in the real-life follow-up care of patients with HVA in the general population.

## Limitations

Our study has several limitations. First, there are inherent limitations of a medical record database, which include reliance on coding to identify cases of anaphylaxis and documentation of an insect sting. Additionally, our study is also limited by the inherent limitations of a retrospective questionnaire based study: patients had to both remember and accurately report. It is highly likely that many of the respondents were not completely accurate historians, as evidenced by some patients reporting that their last sting reaction leading to an emergency intervention happened before that identified in the emergency medical records. We also had a relatively small sample size, with a total of 126 patients. The small sample size is particularly notable when we look at the number of patients with a grade 1 or grade 4 reaction. Both of these groups have less than 10 individuals.

Finally, there may have been a significant population bias. Although we attempted to capture a representative population of patients with HVA by identifying patients based on emergency medical response center records, the patients included in our study ultimately needed to decide that they wanted to invest the time to participate. In addition, 27% of respondents had received treatment from our clinic, and their decision to respond may have also been driven by their recognition of the physicians conducting the study. Even patients with no relation to UKF may have been motivated by how affected they were by their HVA medical emergency. It is possible that patients who were more concerned about their allergy were more likely to respond, thus, increasing the likelihood that respondents had sought follow-up.

On the other hand, by asking patients directly we were able to capture information about care received from multiple providers, as well as to gain insight as to why patients chose not to receive VIT. Our findings can be used as an indicator of the follow-up care that patients in Germany currently receive after the initial emergency treatment of HVA.

## Conclusion

To our knowledge, this is the first study to describe outcomes of allergy follow-up among patients with stinging insect anaphylaxis in Germany. While our study shows higher rates of emergency medication prescription, allergist follow-up, and VIT than previous studies in other countries, there is still plenty of room for improvement. First, there is an information deficit on the relevance of HVA and the correct follow-up care among Emergency Response Teams, primary care physicians and patients. This is reflected in the low rates at which patients received allergy identification cards, prescriptions for emergency medications, and recommendations for follow-up during the course of the acute treatment. From our results, it is clear that follow-up with an allergist essentially guarantees that a patient will receive diagnostic testing and information on treatment options and it is evident that a recommendation for follow-up does positively influence the likelihood that a patient will see an allergist. Thus, an important first step in improving follow-up care would be (1) to inform the patient already during the treatment phase of the index event about the need for diagnostic testing and the availability of a treatment that efficiently protects from recurrence of insect sting anaphylaxis and (2) to refer patients to an allergist.

After the index event, follow-up care does not adequately administer preventative measures such as educating patients on sting prevention, ensuring that emergency medications are kept current and encouraging patients to seek follow-up with an allergist. Structured information material on HVA and the recommended follow-up care should be made available for emergency response teams, emergency departments, and primary care physicians to provide the right information to the right patient at the right time.

Finally, a disappointingly high number of patients chose not to undergo VIT despite receiving diagnostics and information about the therapy. Here, more detailed studies with higher patient numbers are required that allow insight into potential hurdles or misconceptions that may prevent patients from receiving and/or accepting the recommended treatment.

## Abbreviations

BK: Bad Krozingen
EAI: Epinephrine auto-injectors
FR: Freiburg
GP: Göppingen
HVA: Hymenoptera venom allergy
UKF: Medical Center Freiburg
VIT: Venom immunotherapy
